# The stability of IRT parameters under several test equating conditions

**DOI:** 10.3389/fpsyg.2025.1652341

**Published:** 2026-01-12

**Authors:** Dominik Weber, Nicolas Becker, Frank M. Spinath, Marco Koch

**Affiliations:** Department of Individual Differences & Psychodiagnostics, Saarland University, Saarbrücken, Germany

**Keywords:** test equating, item linking, test validity, anchor item, item response theory, simulation study

## Abstract

**Introduction:**

It is crucial for researchers and test developers to compare results from different test sets (e. g., re-testing, parallel test forms). To ensure comparability, test sets are often linked using anchor items as a common denominator alongside distinct items. To date, most studies on test equating have been limited in scope, typically comparing only absolute numbers of anchor items or focusing on a single IRT model or equating method. Furthermore, previous research has primarily evaluated the absolute deviation of estimated parameters from true parameters. However, in diagnostic contexts, the correlation between these values is often more relevant for ensuring validity and test fairness. Therefore, the aim of this simulation study was to examine the impact of a broad range of key factors on test equating.

**Methods:**

We evaluated correlations and recovery indices between predefined true values and values estimated through test equating for three IRT parameters (discrimination, difficulty, and ability). To this end, we varied the equating method (MS, MM, MGM, IRF, TRF), the IRT model (2PL vs. 3PL), guessing probability (0.000–0.250), anchor item proportion (5–25%), test set size (20–80 items), and the discrimination parameters of the anchor items. In addition, we used samples of 25–100 individuals to assess equating quality under challenging conditions as well as samples of 500 and 1,000 individuals to reflect adequate modeling conditions.

**Results:**

Low guessing probabilities and high anchor item discrimination parameters strongly improved test equating quality for all three IRT parameters. Recovery of discrimination and ability parameters increased logarithmically with larger test set sizes and higher anchor item proportions, with each of these two factors partially compensating for reductions in the other. While sample sizes below 100 individuals produced inadequate parameter recovery, samples of 100 or 500 individuals were justifiable under certain conditions. However, samples of only 100 individuals carried a slight risk of non-convergence. The choice of the equating method had rather minor effects and the impact of the IRT model was ambivalent.

**Discussion:**

These findings highlight the importance of using distractor-free response formats without any guessing probability, anchor items with high discrimination parameters, and large samples to ensure valid test equating. For individual research and test application purposes, we provide a comprehensive data set covering multiple factor levels and a step-by-step simulation guide.

## 1 Introduction

Test equating comprises a class of methods to ensure the comparability of different test sets when administering them to identical samples is not feasible. Test comparability is a prerequisite for validity and test fairness and is therefore crucial for panel studies or group assessments with different test sets (Battauz, 2013; Cook and Eignor, 1991; Kolen and Brennan, 2014). A typical approach to test equating is the use of anchor items, which place the item parameters of different test sets on a common scale. The goal of this simulation study was to investigate, using an IRT parameter recovery approach, which factors determine how well anchor item-based test equating performs.

### 1.1 Necessity of multiple test sets

Psychological tests must adhere to high psychometric standards to be applied reasonably in diagnostics and research. Test development is therefore an important process, which often proves to be time-consuming. In particular, the development of parallel test sets can require large amounts of effort. However, parallel tests are essential for test repetition and integrity. Especially in performance testing, practice effects in within-subject designs can occur when the same test is applied repeatedly (Hausknecht et al., 2007; Kulik et al., 1984). This can lead to biased results since in the re-test participants may need fewer cognitive resources to solve items which they had not solved at the first measurement (Ackerman, 1987). Given differentially varying increases in the variable of interest, mean test score differences become unrepresentative of real changes in the underlying variable and thus decrease the validity of the test (e.g., Hausknecht et al., 2002). In between-subject designs, when larger samples are studied over a longer timeframe, test material might become public. Under such circumstances, some participants can prepare specifically for the test with the leaked test materials. As a result, it is not clear whether the test assesses true ability or whether the result is biased by factors such as motivation for preparation or economic capability to obtain or purchase materials. These effects make it necessary to create parallel test sets or at least to constantly renew and standardize the items of a test to ensure validity and test fairness.

### 1.2 Challenges in the application of multiple test sets

Creating parallel test sets is a difficult endeavor. Not only must test developers create twice as many items, they also must ensure that the parallel test sets have equal psychometric properties. Failure to provide this equality can result in test sets of different difficulty which produce mean differences between test takers that are unrelated to true differences in the underlying construct. Since this artificially alters the actual ranking order of the test-takers in the construct of interest, test fairness and validity might be impaired. It is therefore mandatory to validate the item pool from which various test sets are generated in a broad sample. Only if the difficulties of all items are measured on a common scale it is possible to determine the ability of a tested individual independently from the respective test set. For this purpose, item response theory (IRT; Birnbaum, 1968; Rasch, 1960) can be applied: in IRT models, ability parameters (e.g., intelligence) and item parameters (e.g., item difficulty) are determined by maximum likelihood estimation based on the results of several participants on several items. There are three common IRT models that contain a different number of parameters. The simplest and most restrictive model is the Rasch model (1PL model) which only takes the item difficulty and the participant's ability into account. The probability *p*_*xi*_ that a participant *x* with the ability θ_*x*_ solves an item *i* with the difficulty *b*_*i*_ is


$$\begin{array}{l} p_{xi}\left(\theta_{x},\;b_{i}\right)=\frac{exp\left(\theta_{x}-b_{i}\right)}{1+exp\left(\theta_{x}-b_{i}\right)}\; \end{array}$$

While the 1PL model assumes for all items the same capacity to discriminate between different able participants, the 2PL model is less restrictive and takes the more usual case of different item discrimination parameters *a*_*i*_ into account:


$$\begin{array}{l} p_{xi}\left(\theta_{x},\;a_{i},\;b_{i}\right)=\frac{exp\left[a_{i}\left(\theta_{x}-b_{i}\right)\right]}{1+exp\left[a_{i}\left(\theta_{x}-b_{i}\right)\right]} \end{array}$$

Finally, the 3PL model further considers guessing probabilities *c*_*i*_ (e.g., for the case that multiple choice items are applied):


$$\begin{array}{l} p_{xi}\left(\theta_{x},\;a_{i},\;b_{i},\;c_{i}\right)=c_{i}+\left(1-c_{i}\right)\frac{exp\left[a_{i}\left(\theta_{x}-b_{i}\right)\right]}{1+exp\left[a_{i}\left(\theta_{x}-b_{i}\right)\right]} \end{array}$$

However, the resulting parameters depend on the sample that has taken the test: in a highly able sample, items are solved by more participants resulting in lower estimates of the item difficulties than in a less able sample. In practice, despite randomization, it is hardly possible to collect samples of exactly equal ability and even if samples could be matched on a broad range of criteria, people might differ in several confounding factors (i.e., how fast they tire). As a result, the estimated parameters are unequally scaled, and the test sets are not comparable (Battauz, 2015). To obtain equally scaled item difficulties, an item pool needs to be completed by a single sample. However, this is only possible in theory. In fact, the longer a test takes, the fewer participants will work on all test items. Dropout and careless responding (e.g., marking response options at random) might occur more frequently which would impair parameter estimates. Furthermore, ethical aspects such as those provided by the ITC guidelines (International Test Commission, 2001), require that a test must not impose unreasonable strain on the test takers.

### 1.3 Anchor items as an approach to test equating

To address this issue, various test equating strategies (Cook and Eignor, 1991; Kolen and Brennan, 2014) have been developed in an effort to establish the comparability of different test sets without the need to assess only one sample that completes all items from an item pool. The most common strategy is the application of anchor items. Anchor items are shared throughout different test sets, which allows the transformation of item parameters from one test set into the scales of other test sets. There are two types of anchor item-based test equating (Battauz, 2015): direct and indirect equating. In direct equating, all test sets contain the same set of anchor items. Based on the anchor items, equating coefficients are determined, that serve to transform the parameters of one test set into the parameters of another test set by linear transformation following the function


$$\begin{array}{l} {\hat{b}}_{2i}=f\left(b_{1i}\right)=A_{1,2}b_{1i}+B_{1,2} \end{array}$$

where ${\hat{b}}_{2i}$ is the estimated difficulty of item *i* in test set 2 with an empirical difficulty *b*_1*i*_ from test set 1. There are five common methods to compute the equating coefficient *A*_1, 2_ and the equating constant *B*_1, 2_: mean-sigma (MS) method, mean-mean (MM) method, mean-geometric mean (MGM) method, item response function (IRF) method, and test response function (TRF) method. The equating coefficient *A*_1, 2_ is computed by


$$\begin{array}{l} A_{1,2}=\frac{\sigma_{b1}}{\sigma_{b2}} \end{array}$$

for the MS method (cf., Kolen and Brennan, 2014; Marco, 1977), by


$$\begin{array}{l} A_{1,2}=\frac{\overset{l_{1,2}}{\underset{i=1}{\sum }}a_{1i}}{\overset{l_{1,2}}{\underset{i=1}{\sum }}a_{2i}} \end{array}$$

for the MM method (cf. Battauz, 2015), and by


$$\begin{array}{l} A_{1,2}={\left(\overset{l_{1,2}}{\underset{i=1}{\prod }}\frac{a_{1i}}{a_{2i}}\right)}^{\frac{1}{l_{1,2}}} \end{array}$$

for the MGM method (cf. Battauz, 2015), where *l* is the number of anchor items, *a*_1*i*_ and *a*_2*i*_ are the empirical discrimination parameters of the anchor items within test set 1 and test set 2, respectively, and σ_b1_ and σ_b2_ are the standard deviations of the item difficulties of test set 1 and test set 2, respectively. The equating constant *B*_1, 2_ is computed by


$$\begin{array}{l} B_{1,2}=\frac{\overset{l_{1,2}}{\underset{i=1}{\sum }}b_{2i}-A_{1,2}\overset{l_{1,2}}{\underset{i=1}{\sum }}b_{1i}}{l_{1,2}} \end{array}$$

where *b*_1*i*_ and *b*_2*i*_ are the empirical difficulties within test set 1 and test set 2, respectively.

The IRF method is based on the Haebara method (Haebara, 1980) and minimizes the following loss function to obtain the equating coefficient *A*_1, 2_ and the equating constant *B*_1, 2_:


$$\begin{array}{c} f\left(A_{1,2},\;B_{1,2}\right)=\\ \frac{1}{2}{\int_{-\infty }^{+\infty }\sum_{i=1}^{l_{1,2}}\left[p_{2i}\left(\theta ,a_{2i},\;b_{2i},c_{2i}\right)-p_{1i}\left(\theta ,a_{1i}^{'},\;b_{1i}^{'},c_{1i}\right)\right]}^{2}h(\theta )d\theta \end{array}$$

where *h*(θ) is the density of a standardized variable,


$$\begin{array}{l} {a^{\prime }}_{1i}=\frac{a_{1i}}{A_{1,2}} \end{array}$$

and


$$\begin{array}{l} {b^{\prime }}_{1i}=A_{1,2}b_{1i}+B_{1,2} \end{array}$$

Finally, the TRF method minimizes based on the Stocking-Lord method (Stocking and Lord, 1983) the following loss function:


$$\begin{array}{c} \begin{array}{c} f(A_{1,2},\;B_{1,2})=\frac{1}{2}\int_{-\infty }^{+\infty }\\ {\left\{\sum_{i=1}^{l_{1,2}}\left[p_{2i}\left(\theta ;a_{2i},\;b_{2i},c_{2i}\right)-p_{1i}\left(\theta ;a_{1i}^{'},\;b_{1i}^{'},c_{1i}\right)\right]\right\}}^{2}h\left(\theta \right)d\theta \end{array} \end{array}$$

While direct equating is easy to administer, it is only suitable in situations when all test sets are administered to the samples simultaneously. In case of a delay between test sessions, item leakage becomes possible, and the utility of the anchor items might deteriorate. This may result in invalid parameter estimates for the rest of the item set. In contrast, indirect equating proves to be less susceptible to such biases since not every test set contains the same anchor items, but the sets are linked via chains of different anchor items. To this end, based on the direct equating coefficients, a linear function of the form


$$\begin{array}{l} {\hat{b}}_{3i}=f\left(b_{1i}\right)=A_{c}b_{1i}+B_{c}\; \end{array}$$

can be set up to transform the empirical difficulty parameters of test set 1 into the scale of test set 3 with the equating coefficient


$$\begin{array}{l} A_{c}=A_{1,2,\ldots ,m}=\overset{m=3}{\underset{j=2}{\prod }}A_{j-1,\;j}=A_{1,2}A_{2,3} \end{array}$$

and the equating constant


$$\begin{array}{l} B_{c}=B_{1,2\ldots ,m}=\overset{m=3}{\underset{j=2}{\sum }}B_{j-1,j}A_{2,\ldots ,m}=B_{1,2}A_{2,\ldots ,m}+B_{2,3}A_{2,\ldots ,m} \end{array}$$

where


$$\begin{array}{l} A_{2,\ldots ,m}=\overset{m=3}{\underset{h=j+1}{\prod }}A_{h-1,h}=A_{2,3} \end{array}$$

(cf. Battauz, 2015). Based on simulated data, Battauz (2017) found that the choice of the equating method (MM, MGM, IRF, TRF) does not affect the parameter estimates. However, there were some limitations: The IRT modeling was based on the 2PL model only and did not consider the possible effect of the guessing probability. Further, the absolute difference between the estimated and true values was taken as the outcome variable. However, in some cases, the correlation between the estimated and true values (i.e., the stability of the IRT parameters) might be more important in terms of validity and test fairness (e.g., student selection tests where the individual result in comparison to the results of the other participants is crucial). Finally, the anchor item proportion did not vary since the test set size was fixed to 40 items and the number of anchor items to five. It is plausible that the anchor item proportion might affect the accuracy of test equating since more anchor items contain more information.

### 1.4 Number of anchor items

Obviously, if only one anchor item was used, the resulting parameter estimates are prone to bias and lack stability. It is commonly assumed that the more anchor items are applied, the better test equating works. However, apart from some rules of thumb, only few empirical studies have addressed the question of how many anchor items are required for test equating so far. Coffman (1971) recommends adopting 20 items of the original test set when creating a new test set. Alternatively, test sets should share at least 20% of the total item set if this results in more than 20 anchor items. Hills et al. (1988) compared the results of test equating based on six anchor item sets of different sizes, while the number of unshared items was fixed at 45. Test equating worked well based on sets of 10, 15, 20, 25, and 30 anchor items, whereas a set of five anchor items failed to produce satisfactory results. Yang and Houang (1996) compared results based on anchor item sets of 12, 20, and 30 items, while the total test set size was 60 items in each case. They concluded that all three anchor item sets led to similar results. Finally, Sinharay and Holland (2007) compared in an extensive simulation study the equating results of different test set sizes with different anchor item numbers. They showed that equating performs better for larger test sets with more anchor items. However, they fixed the combinations of test set size and anchor item number and did not recombine them, resulting in only three factor levels with nearly the same relative anchor item proportion (41.67–44.87%). Further, the smallest set consisted of 20 anchor items. For tests with large numbers of items and short item processing times, these recommendations may be feasible. However, if tests are short, containing, for instance, 30 items that require a longer processing time (e.g., figural matrix tests where an item puts high cognitive demands on the participants), then different test sets might consist of more shared than distinct items. Under such circumstances, the goal of creating different test sets to prevent test manipulation is prone to fail.

### 1.5 Impact of the anchor item discrimination parameter

In addition to the number of anchor items used, there has been research on the impact of the discrimination parameters of the anchor items: in the context of differential item functioning (DIF), Lopez Rivas et al. (2009) varied the discrimination parameters of the anchor items. In a simulation study, they generated sets of one, three and five anchor items with either low (*a* = 0.60) or high (*a* = 1.20) discrimination parameters. They found an advantage of the higher discriminating anchor items in the power of detecting predefined items with DIF. Further, they mentioned in terms of saturation effect diminishing gains in power when adding items. Similarly, Meade and Wright (2012) recommend employing up to five invariant items with large discrimination parameters. Finally, MacCallum et al. (1999) found that higher communalities (which can be interpreted as a factor analytical analog to IRT discrimination parameters) facilitate accurate recovery of population parameters.

### 1.6 Impact of the sample size

Previous studies comparing equating methods have largely been conducted under idealized simulation conditions, such as large sample sizes and samples with similar latent means and variances. It is generally recommended that stable IRT parameter estimation requires sample sizes of *n* = 500 for 2PL models and *n* = 1,000 for 3PL models (e.g., De Ayala, 2013). However, for samples of 500 (1,000) individuals, the expected sampling error in the sample mean is 0.04 (0.03), and in the sample variance it is 0.06 (0.04). This degree of variation is comparatively small and makes it easy for the equating methods to recover the latent trait. To meaningfully challenge equating methods and determine their performance under realistic instability, reduced sample sizes of fewer than 500 (1,000) individuals with differing latent means and variances would be necessary. Such instability would be expected to increase the importance of the number of anchor items, as they would help to better capture the variation in trait means and variances.

### 1.7 The present study

To our best knowledge, there is no systematic state-of-the-art analysis that determines simultaneously the effects of varying characteristics of the previously discussed key influences on test equating, including challenging conditions such as small sample sizes. In order to answer this question as validly as possible and to provide recommendations on required test equating characteristics, very large samples are necessary. To address this issue, simulation studies are an appropriate methodological approach. There are several advantages using simulation studies (Hallgren, 2013; Morris et al., 2019): they prove to be more economical than empirical studies because they do not take time to acquire participants and collect data. In addition, simulation studies are particularly convenient for comparing statistical methods: if, for example, the true value of a parameter is already known, it is possible to find out which statistical method can be applied to estimate it with the highest accuracy. Since the goal of this study was to ensure the comparability of different test sets, we used simulated data to examine the stability of IRT parameters (i.e., discrimination parameter, item difficulty and ability parameter) under various test equating conditions. In this study, we defined stability as the correlation and deviation between the true (i.e., predefined) parameters and the parameters estimated through test equating in terms of parameter recovery. We investigated the influence of (1) the equating method, (2) the IRT model and the height of guessing probability, (3) the anchor item proportion and test set size, and (4) the sample size. To address these research questions, we had nine hypotheses (H1 to H9):

(H1) The choice of the equating method does not affect the stability of the IRT parameters (i.e., discrimination parameter, item difficulty and ability parameter). This would be in line with Battauz (2017) who showed similar outcomes of several test equating methods for the discrimination parameter and item difficulty.

(H2) The IRT model (2PL vs. 3PL) has an influence on the stability of the parameters. Since the 3PL model takes the guessing probability into account we expect it to result in better parameter recovery than the 2PL model.

(H3) The height of the guessing probability *c* has a negative influence on the stability since responding to items by random does not reflect participants' true ability. We would expect a linear decrease of the guessing probability to result in a logarithmic increase in stabilities in terms of a saturation effect. However, in practice the guessing probability does not decrease linearly by adding further distractors to an item but follows the function *c* = f(*d*) = *d*^−1^ with *d* = number of distractors (i.e., *c*_*d* = 2_ = 0.500, *c*_*d* = 3_ = 0.333, *c*_*d* = 4_ = 0.250 etc.). Since this function and the logarithmic saturation function multiplicate approximately to a linear function we expect a linear decrease of the guessing probability to result in a linear increase of the stabilities.

(H4) Linear increases of the proportion of anchor items within a test set result in logarithmic increases of the stabilities. The anchor items serve as a common denominator for different test sets. Based on their parameters, the parameter estimates of the remaining items are computed. As parameter estimates are more robust the more reference values are used for estimation, we expected the stabilities of the IRT parameters to increase along with the anchor item proportion. We assumed that the estimation gains in stability especially when only few anchor items are included in the test sets. If the test sets already contain many anchor items, additional anchor items should result in less additional stability, following the law of diminishing marginal utility (Marshall and Guillebaud, 1961). Lopez Rivas et al. (2009) found initial evidence for this diminishing gain when adding anchor items. Therefore, we expected a logarithmic trend for the increase of stability with increasing anchor item proportion.

(H5) Linear increases of the test set size result in logarithmic increases of the stabilities. If only a few anchor items are used, it is possible that some might coincidentally be extremely easy or extremely difficult and thus not representative of the total set of items. If the parameters of the remaining items are estimated based on such anchor items, it can be assumed that they are less stable compared to representative items. If, on the other hand, more anchor items are used, random extreme parameters average out, resulting in higher stability of the parameters. Fixing the anchor item proportion to a constant value (e.g., 10%), the absolute number of anchor items varies (e.g., 2 vs. 10) depending on the test set size (e.g., 20 vs. 100). Sinharay and Holland (2007) found an advantage of larger test sets with a similar anchor item proportion to smaller test sets. Thus, we hypothesized that the stability of the IRT parameters increases with the test set size. Analogously to H3, we expected in terms of the law of diminishing utility a decreasing gain in stability with increasing test set size, and thus increasing amount of anchor items. Consequently, we expected a logarithmic trend of the test set size as well.

(H6) There is an interaction effect between the factors test set size and anchor item proportion. Based on H5 and H6, we assumed that the differences between the anchor item proportions decrease with increasing test set size due to a saturation effect based on the high absolute number of anchor items in large test sets. Therefore, we expected the absolute anchor item number to be a substantial predictor of the stability.

(H7) Higher discrimination parameters of the anchor items result in higher correlations and smaller deviations between the true IRT parameters and the parameters estimated through test equating. The discrimination parameter is the capability of an item to differentiate between individuals with different ability parameters. Lopez Rivas et al. (2009) and Meade and Wright (2012) found evidence for the advantage of anchor items with high discrimination parameters in detecting items with DIF. Transferred to test equating, we expected the estimation of the IRT parameters to be more accurate based on anchor items with high discrimination parameters.

(H8) Larger sample sizes result in a logarithmic increase of the stability of IRT parameters. Typically, sample sizes of *n* = 500 (*n* = 1,000) individuals are recommended for 2PL (3PL) models. When sample sizes fall below these recommendations, models should fail to converge more frequently and negatively affect parameter recovery, as increased sampling error and single outliers may bias the estimated latent traits. Consistent with the effects of anchor item proportion and the test set size, we expected a saturation effect when increasing sample size.

(H9) There is an interaction effect between sample size and anchor item proportion. In small samples, the characteristics of the anchor items are estimated with lower precision. Hence, a larger number of anchor items could help average out this reduced precision. In contrast, in large samples, the characteristics of single anchor items are estimated more precisely, so increasing the anchor item proportion would provide comparatively less additional precision.

## 2 Methods

### 2.1 Sample

We simulated samples of five different sizes: to examine test equating under challenging conditions, we generated samples of *n* = 25, 50 and 100 individuals. To assess performance under adequate IRT modeling conditions, we additionally generated samples of *n* = 500 and 1,000 individuals. In each iteration, the latent means of three samples were drawn from normal distribution with the means *M*_θ_ = −0.50, 0.00, and +0.50, and with the standard deviations *SD*_θ_ = 0.80, 1.00, and 1.20 in order to manipulate heterogeneity in latent means and variances.

### 2.2 Independent variables

To address H1, we varied the equating method. Test equating was conducted using the MS, MM, MGM, IRF and TRF methods. To address H2, we applied two IRT models: 2PL model (varying discrimination parameters) and the 3PL model (additionally accounting for the guessing probability). To address H3, we manipulated the guessing probability: we included the most common cases of no guessing probability (e.g., in a test with distractor-free response formats) and of four (*c*_*d* = 4_ = 0.250), six (*c*_*d* = 6_ = 0.167), and eight (*c*_*d* = 8_ = 0.125) distractors. To address H4 to H6, we manipulated both the proportion of anchor items within the test sets and the test set size: proportions of 5, 10, 15, 20 and 25% were implemented within test sets of 20, 40, 60, and 80 items. Indirect equating was used to achieve comparability across three test sets. To ensure equal conditions for all factor combinations, within one iteration the same samples were taken for each of them.

### 2.3 Simulation procedure and test equating

**Figure 1:**
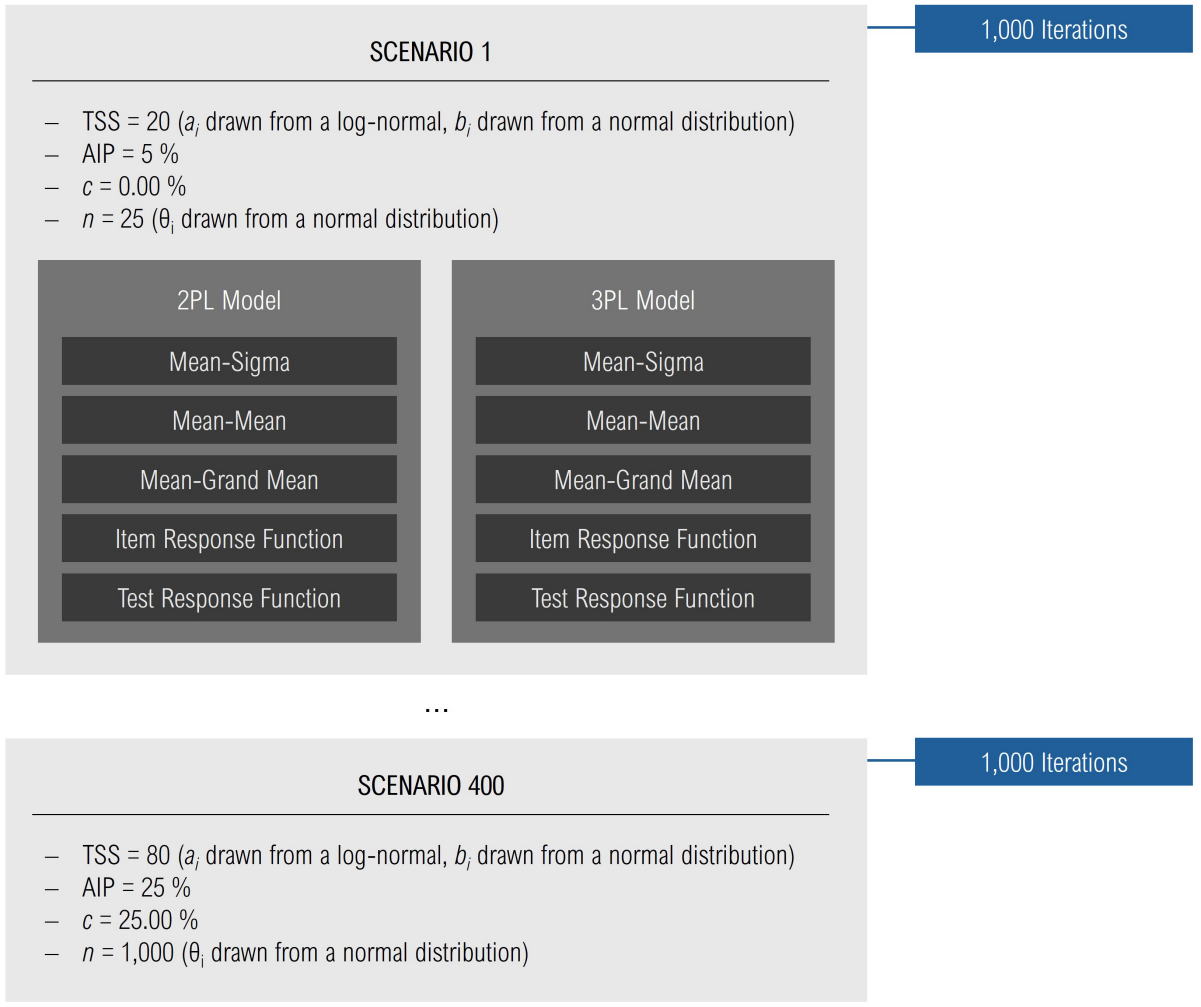
Independent variables and their dependencies within the simulation. The figure shows the six independent variables: TSS, test set size; AIP, anchor item proportion; *c*, guessing probability; *n*, sample size; IRT model, and equating method. 400 scenarios as a combination of test set size × anchor item proportion × guessing probability × sample size were simulated. Within a scenario, discrimination and difficulty parameters of the items and ability parameters of the simulees were drewn. Based on these parameters, the simulees' responses to the items were generated. From these responses, in each scenario IRT parameters were estimated with a 2PL as well as with a 3PL model. With the resulting test-specific parameters, five equating methods were applied to achieve parameters of a common scale.

We simulated data for 400 different equating scenarios, corresponding to all combinations of the factors test set size × anchor item proportion × sample size × guessing probability. Within each scenario, the “true” discrimination parameters *a*_*i*_ were drawn from a log-normal distribution with *M*_*a*_ = 1.00 and *SD*_*a*_ = 0.20 to ensure positive values, while item difficulties *b*_*i*_ were drawn from a normal distribution (*M*_*b*_ = 0.00, *SD*_*b*_ = 1.00). For each scenario, we conducted 1,000 iterations, meaning that the true IRT parameters were newly generated 1,000 times. Participants' responses were simulated based on these item difficulties and ability parameters. We used the *R* (R Core Team, 2021) package *catIrt* (Nydick, 2014), which produces binary outcomes indicating whether each item was solved (1) or not (0). Item parameters were then estimated on a test set-specific scale first by means of 2PL and second by means of 3PL modeling using the *mirt* package (Chalmers, 2012). Based on the anchor items, the five test equating methods were performed with the package *equateMultiple* (Battauz, 2021). This resulted in a common item parameter scale across the three linked test sets. Figure 1 provides an overview of the independent variables and their dependencies.

### 2.4 Dependent variables

To quantify the stability of the IRT parameters, we computed common indices of parameter recovery: First, we calculated the correlations between the true and the estimated values for each IRT parameter as an *association-based recovery index*. Although focusing on the correlation is informative in diagnostic settings in which an individual's test result is interpreted relative to other test takers (e.g., in student selection tests), this criterion can mask distortions arising from inherent identification issues in IRT models (as discussed in Noventa et al., 2024): Because IRT models allow multiple equivalent parameterizations, systematic underestimation (overestimation) of discrimination parameters can lead to an inflation (deflation) of the latent trait variance. Likewise, downward (upward) bias in item difficulties can shift the ability estimates downward (upward). As a result, correlations between true and estimated values remain high even when the ability parameter can no longer be interpreted as a standardized score. Therefore, in addition to the correlation as an association-biased recovery index, we computed bias, Mean Absolute Error (MAE), and Standard Error of Estimate (SEE) as *error-based recovery indices*. Finally, we calculated Root Mean Square Error (RMSE), which captures bias, absolute error, and the linearity between true and estimated values (Roberts and Laughlin, 1996).

### 2.5 Statistical analysis

Due to the large amount of simulated data, extreme values that are not plausible under real conditions were expected. Therefore, for each iteration, the values in the upper and lower 2.5% quantiles were removed, and the inner 95% of the 1,000 values were retained for analysis. In line with our hypotheses, we estimated a linear mixed model (LMM) for each parameter recovery index with the following fixed effects: equating method (linear), IRT model (linear), guessing probability (linear), anchor item proportion (logarithmic), test set size (logarithmic), interaction effect of the anchor item proportion (logarithmic) and the test set size (logarithmic), discrimination parameter of the anchor items (linear), samples size (logarithmic), and interaction effect of the sample size (logarithmic) and the anchor item proportion (logarithmic). To account for repeated measurements and for an imbalance of the number of converged iterations between models, we additionally specified random intercepts for the scenario (test set size × anchor item proportion × sample size × guessing probability) as well as for the iteration within a scenario (1 to 1,000). This resulted in the following model equation (illustrated for RMSE of the item difficulty):


$$\begin{array}{r} {RMSE_{b}}_{isr}\;\sim \;b_{0}+b_{1}^{T}\cdot method_{i}+b_{2}^{T}\cdot model_{i}+b_{3}\cdot \\ c_{i}+b_{4}\cdot \ln\left(AIP_{i}\right)+b_{5}\cdot \ln\left(TSS_{i}\right)+b_{6}\cdot \ln\left(AIP_{i}\right)\cdot \ln\left(TSS_{i}\right)+b_{7}\\ \cdot MAA_{i}+b_{8}\cdot \ln\left(n_{i}\right)+b_{9}\cdot \ln\left(n_{i}\right)\cdot \ln\left(AIP_{i}\right)+u_{s}+v_{sr}+\varepsilon_{isr} \end{array}$$

with *c* = guessing probability, ln = natural logarithm, AIP = anchor item proportion, TSS = test set size, MAA = mean anchor item discrimination parameter, *n* = sample size, *u*_*s*_ = random intercept of the scenario (combination of test set size × anchor item proportion × sample size × guessing probability), *v*_*sr*_ = random intercept of an iteration within a scenario, and ε_*isr*_ = residual error term. Data analyses were conducted using the *R* packages *lme4* (Bates et al., 2015) and *lmerTest* (Kuznetsova et al., 2017). Given the large number of significance tests, we corrected for multiple testing: we applied Benjamini-and-Hochberg correction (Benjamini and Hochberg, 1995) covering all model tests to control the False Discovery Rate (FDR) at *q* = 0.050, ensuring that the proportion of false positives among significant results remained below 5%. Our simulations, the resulting data set, and the *R* script for analyzing the data are provided in our OSF repository: https://osf.io/np7k9/?view_only=dcb2b2b1bb02426e95b882f48378aa81.

### 2.6 *Post-hoc* analyses

Even when average parameter recovery is satisfactory, results are only meaningful if model convergence occurs with sufficient probability. Hence, as a *post-hoc* analysis, we examined the conditions under which model convergence is likely. We therefore computed the mean convergence rate (CR = [0, 1]) and the mean number of converged test sets [0, 3] per scenario (test set size × anchor item proportion × sample size × guessing probability) and tested the effects of the IRT model, the guessing probability, the anchor item proportion, and the sample size for significance using a LMM. Since each scenario (i.e., the same sample) contributed two convergence statistics (one regarding 2PL, one regarding 3PL model), we specified the scenario as a random intercept. This resulted in the following model equation:


$$\begin{array}{c} convergence_{is}\;\sim \;b_{0}+b_{1}^{T}\cdot model_{i}+b_{2}\cdot c_{i}+b_{3}\cdot AIP_{i}+b_{4}\cdot \\ TSS_{i}+b_{5}\cdot n_{i}+u_{s}+\varepsilon_{is} \end{array}$$

where *c* = guessing probability, AIP = anchor item proportion, TSS = test set size, *n* = sample size, *u*_*s*_ = random intercept of the scenario, and ε_*isr*_ = residual error term. As in the main analysis, we applied Benjamini-and-Hochberg correction to control the FDR at *q* = 0.050.

## 3 Results

### 3.1 Descriptive statistics

Across all equating scenarios, mean correlation between true and estimated discrimination parameters was *r* = 0.55. Mean bias was 0.46, mean RMSE was 1.25, mean MAE was 0.80, and mean SEE was 1.11. For item difficulties, mean correlation was *r* = 0.81, mean bias was 0.24, mean RMSE was 8.11, mean MAE was 4.68, and mean SEE was 7.39. For ability parameters, mean correlation was *r* = 0.83, mean bias was −0.16, mean RMSE was 8.01, mean MAE was 4.62, and mean SEE was 6.90. Tables 1–3 present the mean parameter recovery indices across predictor levels.

**Table 1:** Descriptive statistics of the parameter recovery criterions for the discrimination parameter.

**Factor**	**Level**	***r***	**Bias**	**RMSE**	**MAE**	**SEE**
Method	MS	0.54	0.47	1.21	0.81	1.07
	MM	0.56	0.66	1.55	0.97	1.37
	MGM	0.55	0.36	1.02	0.70	0.89
	IRF	0.54	0.37	1.18	0.73	1.05
	TRF	0.54	0.43	1.31	0.81	1.17
Model	2PL	0.51	0.08	0.75	0.52	0.63
	3PL	0.59	0.85	1.79	1.10	1.62
*c*	0.000	0.68	0.20	0.58	0.39	0.50
	0.125	0.54	0.39	1.15	0.73	1.02
	0.167	0.51	0.51	1.42	0.90	1.27
	0.250	0.47	0.74	1.89	1.20	1.68
AIP	5%	0.51	0.94	1.82	1.33	1.60
	10%	0.55	0.48	1.31	0.83	1.16
	15%	0.56	0.33	1.10	0.66	0.97
	20%	0.56	0.28	1.03	0.61	0.92
	25%	0.56	0.26	1.01	0.58	0.91
TSS	20	0.47	1.11	2.29	1.55	2.04
	40	0.55	0.33	1.08	0.67	0.95
	60	0.58	0.19	0.82	0.49	0.73
	80	0.60	0.12	0.70	0.42	0.62
*n*	25	0.20	3.55	7.00	4.22	6.41
	50	0.33	0.47	1.45	0.93	1.27
	100	0.47	0.05	0.54	0.40	0.45
	500	0.73	−0.07	0.21	0.17	0.15
	1000	0.79	−0.07	0.17	0.14	0.12
Total	–	0.55	0.46	1.25	0.80	1.11

The table shows the means of recovery parameters (in columns) regarding discrimination parameter depending on several predictors (in rows). MS, mean-sigma method; MM, mean-mean method; MGM, mean-geometric mean method; IRF, item response function method; TRF, test-response function method; *c*, guessing probability; AIP, anchor item proportion; TSS, test set size; *n*, sample size; *r*, correlation between true and estimated parameters; RMSE, root mean square error; MAE, mean absolute error; SEE, standard error of estimate.

**Table 2:** Descriptive statistics of the parameter recovery criterions for the item difficulty.

**Factor**	**Level**	***r***	**Bias**	**RMSE**	**MAE**	**SEE**
Method	MS	0.82	0.29	15.61	9.61	14.07
	MM	0.79	0.25	7.14	4.59	6.34
	MGM	0.81	0.14	4.64	3.08	3.84
	IRF	0.82	0.17	4.44	1.91	4.29
	TRF	0.81	0.36	8.42	3.99	8.17
Model	2PL	0.79	0.52	10.33	6.07	9.28
	3PL	0.83	−0.05	5.77	3.21	5.41
*c*	0.000	0.88	0.69	4.17	2.29	3.83
	0.125	0.81	0.28	7.16	4.01	6.52
	0.167	0.79	−1.87	9.27	5.08	8.75
	0.250	0.76	1.94	11.96	7.42	10.57
AIP	5%	0.83	0.22	15.74	9.77	14.37
	10%	0.83	0.26	6.66	3.97	6.05
	15%	0.81	0.77	6.86	3.79	6.19
	20%	0.80	0.09	5.44	2.75	5.09
	25%	0.78	−0.15	5.86	3.13	5.29
TSS	20	0.79	−0.59	8.48	5.47	7.60
	40	0.82	1.16	13.69	8.04	12.41
	60	0.82	−0.29	4.07	2.17	3.78
	80	0.82	0.74	5.82	2.73	5.48
*n*	25	0.42	5.38	31.46	18.89	28.33
	50	0.63	−1.13	18.53	10.22	17.25
	100	0.82	−0.12	3.53	1.94	3.27
	500	0.97	−0.18	0.53	0.38	0.40
	1000	0.98	−0.19	0.36	0.29	0.22
Total	–	0.81	0.24	8.11	4.68	7.39

The table shows the means of recovery parameters (in columns) regarding item difficulty depending on several predictors (in rows). MS, mean-sigma method; MM, mean-mean method; MGM, mean-geometric mean method; IRF, item response function method; TRF, test-response function method; *c*, guessing probability; AIP, anchor item proportion; TSS, test set size; *n*, sample size; *r*, correlation between true and estimated parameters; RMSE, root mean square error; MAE, mean absolute error; SEE, standard error of estimate.

**Table 3:** Descriptive statistics of the parameter recovery criterions for the ability parameter.

**Factor**	**Level**	***r***	**Bias**	**RMSE**	**MAE**	**SEE**
Method	MS	0.81	0.87	23.06	12.25	20.60
	MM	0.80	−0.26	7.06	4.51	5.64
	MGM	0.81	−0.40	5.04	3.24	4.05
	IRF	0.88	−0.15	1.20	0.83	1.07
	TRF	0.87	−0.88	3.09	1.96	2.59
Model	2PL	0.83	0.73	10.14	5.71	8.75
	3PL	0.84	−1.08	5.76	3.47	4.95
*c*	0.000	0.88	0.07	2.81	1.85	2.37
	0.125	0.83	−0.16	4.66	3.01	3.81
	0.167	0.82	−2.03	8.93	5.06	7.73
	0.250	0.79	1.57	15.96	8.71	13.98
AIP	5%	0.82	−1.07	24.98	13.13	22.14
	10%	0.83	−0.42	5.97	3.61	5.12
	15%	0.84	0.53	3.82	2.62	3.07
	20%	0.84	0.13	2.46	1.68	2.01
	25%	0.84	0.05	2.88	2.09	2.19
TSS	20	0.74	−2.02	10.58	6.37	8.94
	40	0.83	1.16	15.95	8.50	14.12
	60	0.87	−0.11	2.20	1.49	1.82
	80	0.89	0.52	2.49	1.65	2.01
*n*	25	0.62	2.90	40.19	21.82	35.18
	50	0.75	−2.25	14.50	8.66	12.10
	100	0.84	−0.03	2.23	1.47	1.89
	500	0.91	0.00	0.56	0.42	0.53
	1000	0.92	−0.06	0.59	0.43	0.56
Total	–	0.83	−0.16	8.01	4.62	6.90

The table shows the means of recovery parameters (in columns) regarding ability parameter depending on several predictors (in rows). MS, mean-sigma method; MM, mean-mean method; MGM, mean-geometric mean method; IRF, item response function method; TRF, test-response function method; *c*, guessing probability; AIP, anchor item proportion; TSS, test set size; *n*, sample size; *r*, correlation between true and estimated parameters; RMSE, root mean square error; MAE, mean absolute error; SEE, standard error of estimate.

### 3.2 Effects on discrimination parameters

For better readability, in the following we primarily report the effects on correlation and RMSE, in particular how they increase or decrease when one of the predictors changes. Tables 4–6 show the results of the LMM analysis for each parameter recovery criterion of each IRT parameter. The equating method significantly predicted the correlation between the true and estimated discrimination parameter, although the effect was comparatively small: switching from the MS to the MM method increased the correlation by Δ*r* = 0.01. Error-based recovery indices were significantly lower when using the MGM method compared with the other methods. Switching from the 2PL to the 3PL model increased the correlation of the discrimination parameters substantially (Δ*r* = 0.07), although error-based recovery indices deteriorated (e.g., ΔRMSE = 1.17). Increasing the guessing probability strongly impaired parameter recovery (Δ*r* = −0.08 and ΔRMSE = 0.73 for an increase of Δ*c* = 0.10). Recovery of discrimination parameters improved with larger anchor item proportions, test set sizes, and sample size (Δ*r* = 0.02 | 0.05 | 0.11 and ΔRMSE = −0.39 | −0.84 | −0.85 when doubling anchor item proportion | test set size | sample size). However, there was a significant interaction between anchor item proportion and test set size: the effect of increasing the anchor item proportion diminished as test set size grew, *b*_*r*_ = −0.02, *t*_(386.71)_ = −9.93, *p* < 0.001 and *b*_*RMSE*_ = 0.47, *t*_(394.40)_ = 3.29, *p* = 0.002. A similar diminishing effect occurred for the interaction between anchor item proportion and sample size with respect to the error-based recovery indices, *b*_*RMSE*_ = 0.45, *t*_(399.43)_ = 3.31, *p* = 0.002. Finally, higher discrimination parameters of the anchor items improved association-based recovery (Δ*r* = 0.11 for an increase of Δ*M*_*a*_ = 0.10) but deteriorated the error-based recovery indices (e.g., ΔRMSE = 0.32).

**Table 4:** Results of the linear mixed models on the discrimination parameter.

**Predictor**	***b_1_***	***df***	***t***	***p***
***r***				
MM vs. MS	0.01	30,86,415.90	14.53	< 0.001
MGM vs. MS	0.00	30,86,255.16	2.73	0.011
IRF vs. MS	−0.01	30,86,559.83	−26.77	< 0.001
TRF vs. MS	−0.01	30,86,763.99	−25.15	< 0.001
Model	0.07	31,06,943.70	247.40	< 0.001
*c*	−0.77	389.77	−40.75	< 0.001
ln(AIP)	0.02	383.99	10.75	< 0.001
ln(TSS)	0.03	386.57	20.10	< 0.001
ln(AIP) × ln(TSS)	−0.02	386.71	−9.93	< 0.001
*M_*a*_*	0.01	32,98,326.09	41.58	< 0.001
ln(*n*)	0.21	392.34	130.17	< 0.001
ln(AIP) × ln(*n*)	0.01	392.53	5.01	< 0.001
**Bias**				
MM vs. MS	0.27	31,00,436.29	6.86	< 0.001
MGM vs. MS	−0.04	30,99,899.20	−0.96	0.404
IRF vs. MS	0.00	31,00,858.03	0.11	0.935
TRF vs. MS	0.07	31,01,602.38	1.75	0.112
Model	0.83	31,47,224.42	33.54	< 0.001
*c*	3.28	397.03	3.21	0.003
ln(AIP)	−0.27	392.50	−2.87	0.008
ln(TSS)	−0.43	394.61	−4.57	< 0.001
ln(AIP) × ln(TSS)	0.35	394.72	3.79	< 0.001
*M_*a*_*	0.14	15,96,633.45	9.28	< 0.001
ln(*n*)	−0.82	399.28	−9.31	< 0.001
ln(AIP) × ln(*n*)	0.37	399.39	4.18	< 0.001
**RMSE**				
MM vs. MS	0.48	3,099,729.19	7.94	< 0.001
MGM vs. MS	−0.06	30,99,209.82	−0.92	0.423
IRF vs. MS	0.16	31,00,140.84	2.67	0.012
TRF vs. MS	0.31	31,00,859.43	5.10	< 0.001
Model	1.17	31,45,874.97	30.32	< 0.001
*c*	7.30	396.87	4.68	< 0.001
ln(AIP)	−0.32	392.03	−2.27	0.036
ln(TSS)	−0.64	394.28	−4.49	< 0.001
ln(AIP) × ln(TSS)	0.47	394.40	3.29	0.002
*M_*a*_*	0.23	16,44,957.43	9.63	< 0.001
ln(*n*)	−1.63	399.31	−12.13	< 0.001
ln(AIP) × ln(*n*)	0.45	399.43	3.31	0.002
**MAE**				
MM vs. MS	0.24	31,00,153.41	6.31	< 0.001
MGM vs. MS	−0.04	30,99,627.08	−0.91	0.429
IRF vs. MS	0.03	31,00,566.91	0.78	0.495
TRF vs. MS	0.12	31,01,295.29	3.11	0.003
Model	0.66	3146558.87	26.59	< 0.001
*c*	4.38	397.40	4.11	< 0.001
ln(AIP)	−0.29	393.11	−3.00	0.005
ln(TSS)	−0.48	395.11	−4.92	< 0.001
ln(AIP) × ln(TSS)	0.37	395.21	3.84	< 0.001
*M_*a*_*	0.16	16,25,491.06	10.79	< 0.001
ln(*n*)	−0.95	399.49	−10.37	< 0.001
ln(AIP) × ln(*n*)	0.39	399.60	4.25	< 0.001
**SEE**				
MM vs. MS	0.43	30,99,813.55	7.15	< 0.001
MGM vs. MS	−0.05	30,99,292.63	−0.87	0.447
IRF vs. MS	0.16	31,00,227.88	2.66	0.013
TRF vs. MS	0.29	31,00,949.01	4.86	< 0.001
Model	1.11	31,46,002.31	28.85	< 0.001
*c*	6.66	396.41	4.49	< 0.001
ln(AIP)	−0.28	391.24	−2.05	0.060
ln(TSS)	−0.57	393.64	−4.21	< 0.001
ln(AIP) × ln(TSS)	0.44	393.77	3.23	0.003
*M_*a*_*	0.21	16,40,621.76	9.02	< 0.001
ln(*n*)	−1.50	399.07	−11.73	< 0.001
ln(AIP) × ln(*n*)	0.40	399.19	3.13	0.003

The table shows the results of five linear mixed models regressing parameter recovery indices on several predictors. MM, mean-mean method; MS, mean-sigma method; MGM, mean-geometric mean method; IRF, item response function method; TRF, test-response function method; *c*, guessing probability; ln, natural logarithm; AIP, anchor item proportion; TSS, test set size; *M*_*a*_, mean anchor item discrimination parameter; *n*, sample size; *df* , degrees of freedom; *r*, correlation between true and estimated parameters; RMSE, root mean square error; MAE, mean absolute error; SEE, standard error of estimate. The predictors AIP, TSS, and *n* were standardized. To correct for multiple testing, *p* values of the Tables 4–6 have been Benjamini-and-Hochberg adjusted (*q* = 0.050).

**Table 5:** Results of the linear mixed models on the item difficulties.

**Predictor**	***b_1_***	***Df***	***t***	***p***
***r***				
MM vs. MS	−0.04	30,82,584.13	−110.19	< 0.001
MGM vs. MS	−0.03	30,82,390.16	−73.04	< 0.001
IRF vs. MS	−0.02	30,82,748.64	−43.12	< 0.001
TRF vs. MS	−0.03	30,82,996.70	−84.40	< 0.001
Model	0.01	31,06,585.84	60.13	< 0.001
*c*	−0.57	392.69	−12.40	< 0.001
ln(AIP)	−0.02	392.00	−4.55	< 0.001
ln(TSS)	−0.01	392.30	−1.43	0.208
ln(AIP) × ln(TSS)	−0.02	392.31	−5.06	< 0.001
*M_*a*_*	0.01	31,52,260.96	78.77	< 0.001
ln(*n*)	0.19	392.89	48.83	< 0.001
ln(AIP) × ln(*n*)	0.03	392.92	6.44	< 0.001
**Bias**				
MM vs. MS	0.03	31,10,525.84	0.01	0.989
MGM vs. MS	−0.05	31,09,525.89	−0.02	0.987
IRF vs. MS	−0.08	31,10,468.04	−0.04	0.979
TRF vs. MS	0.11	31,11,680.05	0.06	0.969
Model	−0.47	31,15,284.52	−0.38	0.757
*c*	3.28	212.16	0.37	0.759
ln(AIP)	−0.22	206.35	−0.28	0.815
ln(TSS)	0.40	207.54	0.49	0.674
ln(AIP) × ln(TSS)	−0.78	207.92	−0.97	0.402
*M_*a*_*	0.48	9,38,542.80	0.76	0.502
ln(*n*)	−0.91	236.19	−1.15	0.319
ln(AIP) × ln(*n*)	0.80	236.49	1.02	0.380
**RMSE**				
MM vs. MS	−7.94	31,10,639.54	−2.49	0.021
MGM vs. MS	−10.43	31,09,699.88	−3.27	0.002
IRF vs. MS	−10.47	31,10,834.95	−3.27	0.002
TRF vs. MS	−6.42	31,12,022.96	−2.01	0.064
Model	−3.98	31,21,058.31	−1.95	0.072
*c*	33.76	255.36	2.35	0.030
ln(AIP)	−3.47	249.07	−2.70	0.012
ln(TSS)	−1.12	249.84	−0.87	0.447
ln(AIP) × ln(TSS)	−0.44	250.31	−0.34	0.771
*M_*a*_*	−1.23	9,85,438.80	−1.16	0.316
ln(*n*)	−9.07	286.06	−7.11	< 0.001
ln(AIP) × ln(*n*)	4.20	286.45	3.29	0.002
**MAE**				
MM vs. MS	−4.73	31,09,473.83	−2.42	0.024
MGM vs. MS	−6.23	31,08,492.95	−3.18	0.003
IRF vs. MS	−7.33	31,09,425.72	−3.73	< 0.001
TRF vs. MS	−5.22	31,10,610.93	−2.66	0.013
Model	−2.53	31,12,530.59	−2.02	0.063
*c*	21.86	214.52	2.49	0.021
ln(AIP)	−2.41	209.06	−3.07	0.004
ln(TSS)	−1.12	209.87	−1.42	0.212
ln(AIP) × ln(TSS)	−0.19	210.27	−0.24	0.838
*M_*a*_*	−0.54	9,52,406.83	−0.84	0.463
ln(*n*)	−5.19	239.88	−6.67	< 0.001
ln(AIP) × ln(*n*)	2.96	240.19	3.80	< 0.001
**SEE**				
MM vs. MS	−7.23	31,11,593.03	−2.48	0.021
MGM vs. MS	−9.73	31,10,660.50	−3.33	0.002
IRF vs. MS	−9.12	31,11,874.70	−3.12	0.003
TRF vs. MS	−5.18	31,13,073.64	−1.77	0.107
Model	−3.33	31,25,110.47	−1.79	0.103
*c*	29.66	278.59	2.26	0.037
ln(AIP)	−3.15	271.83	−2.68	0.013
ln(TSS)	−0.89	272.58	−0.76	0.502
ln(AIP) × ln(TSS)	−0.37	273.10	−0.31	0.790
*M_*a*_*	−1.22	9,91,530.85	−1.25	0.278
ln(*n*)	−8.34	312.32	−7.16	< 0.001
ln(AIP) × ln(*n*)	3.77	312.75	3.23	0.003

The table shows the results of five linear mixed models regressing parameter recovery indices on several predictors. MM, mean-mean method; MS, mean-sigma method; MGM, mean-geometric mean method; IRF, item response function method; TRF, test-response function method; *c*, guessing probability; ln, natural logarithm; AIP, anchor item proportion; TSS, test set size; *M*_*a*_, mean anchor item discrimination parameter; *n*, sample size; *df* , degrees of freedom; *r*, correlation between true and estimated parameters; RMSE, root mean square error; MAE, mean absolute error; SEE, standard error of estimate. The predictors AIP, TSS, and *n* were standardized. To correct for multiple testing, *p* values of the Tables 4–6 have been Benjamini-and-Hochberg adjusted (*q* = 0.050).

**Table 6:** Results of the linear mixed models on the ability parameters.

**Predictor**	***b_1_***	***df***	***t***	***p***
***r***				
MM vs. MS	−0.01	30,78,361.28	−69.44	< 0.001
MGM vs. MS	0.00	30,78,014.86	8.00	< 0.001
IRF vs. MS	0.06	30,78,640.52	298.57	< 0.001
TRF vs. MS	0.05	30,79,103.33	261.85	< 0.001
Model	0.00	31,15,886.49	−24.21	< 0.001
*c*	−0.40	393.10	−16.24	< 0.001
ln(AIP)	0.01	392.66	3.10	0.004
ln(TSS)	0.05	392.86	23.31	< 0.001
ln(AIP) × ln(TSS)	0.00	392.87	−2.00	0.066
*M_*a*_*	0.01	23,31,408.34	74.32	< 0.001
ln(*n*)	0.09	393.24	44.09	< 0.001
ln(AIP) × ln(*n*)	0.00	393.25	−1.46	0.198
**Bias**				
MM vs. MS	−1.21	27,78,807.26	−0.52	0.669
MGM vs. MS	−1.26	27,77,160.02	−0.54	0.658
IRF vs. MS	−1.16	27,79,979.15	−0.49	0.674
TRF vs. MS	−1.88	27,82,272.81	−0.80	0.484
Model	−1.60	28,38,743.31	−1.07	0.354
*c*	5.96	190.39	0.50	0.674
ln(AIP)	0.16	184.42	0.15	0.910
ln(TSS)	0.94	186.17	0.87	0.447
ln(AIP) × ln(TSS)	−1.46	186.48	−1.35	0.237
*M_*a*_*	0.88	5,33,197.04	1.11	0.332
ln(*n*)	−0.37	209.74	−0.35	0.727
ln(AIP) × ln(*n*)	0.51	210.00	0.48	0.633
**RMSE**				
MM vs. MS	−17.38	25,93,554.70	−2.75	0.010
MGM vs. MS	−15.22	25,91,553.53	−3.14	0.003
IRF vs. MS	20.84	25,95,165.13	−3.76	< 0.001
TRF vs. MS	18.89	25,98,009.53	−3.41	0.002
Model	−3.44	26,80,529.19	−0.97	0.401
*c*	63.06	189.37	2.12	0.052
ln(AIP)	−8.56	182.98	−3.20	0.003
ln(TSS)	−3.69	185.15	−1.38	0.226
ln(AIP) × ln(TSS)	3.10	185.45	1.16	0.316
*M_*a*_*	−6.26	3,99,128.67	−3.37	0.002
ln(*n*)	−9.93	206.91	−3.79	< 0.001
ln(AIP) × ln(*n*)	11.07	207.16	4.23	< 0.001
**MAE**				
MM vs. MS	−7.32	26,91,559.67	−2.77	0.010
MGM vs. MS	−8.66	26,89,764.28	−3.28	0.002
IRF vs. MS	−10.86	26,92,959.25	−4.10	< 0.001
TRF vs. MS	−9.69	26,95,494.06	−3.66	0.001
Model	−1.73	27,66,805.40	−1.03	0.375
*c*	32.35	196.49	2.30	0.035
ln(AIP)	−4.26	189.99	−3.37	0.002
ln(TSS)	−2.09	192.12	−1.64	0.140
ln(AIP) × ln(TSS)	1.56	192.43	1.23	0.283
*M_*a*_*	−3.16	4,69,530.04	−3.54	0.001
ln(*n*)	−5.47	215.21	−4.41	< 0.001
ln(AIP) × ln(*n*)	5.45	215.47	4.38	< 0.001
**SEE**				
MM vs. MS	−14.27	25,30,624.63	−2.83	0.008
MGM vs. MS	−15.97	25,28,510.19	−3.17	0.003
IRF vs. MS	−18.64	25,32,366.85	−3.69	0.001
TRF vs. MS	−17.07	25,35,386.37	−3.38	0.002
Model	−2.97	26,25,756.35	−0.92	0.423
*c*	56.34	189.52	2.05	0.061
ln(AIP)	−7.75	183.02	−3.14	0.004
ln(TSS)	−3.21	185.30	−1.30	0.258
ln(AIP) × ln(TSS)	2.80	185.60	1.13	0.327
*M_*a*_*	−5.52	3,63,490.10	−3.28	0.002
ln(*n*)	−8.61	206.56	−3.56	0.001
ln(AIP) × ln(*n*)	10.09	206.80	4.17	< 0.001

The table shows the results of five linear mixed models regressing parameter recovery indices on several predictors. MM, mean-mean method; MS, mean-sigma method; MGM, mean-geometric mean method; IRF, item response function method; TRF, test-response function method; *c*, guessing probability; ln, natural logarithm; AIP, anchor item proportion; TSS, test set size; *M*_*a*_, mean anchor item discrimination parameter; *n*, sample size; *df* , degrees of freedom; *r*, correlation between true and estimated parameters; RMSE, root mean square error; MAE, mean absolute error; SEE, standard error of estimate. The predictors AIP, TSS, and *n* were standardized. To correct for multiple testing, *p* values of the Tables 4–6 have been Benjamini-and-Hochberg adjusted (*q* = 0.050).

### 3.3 Effects on item difficulties

The choice of equating method significantly predicted also recovery indices of the item difficulties: The MM method led to slightly higher correlations between true and estimated item difficulties (Δ*r* = 0.01), and especially the MGM (ΔRMSE = −10.43) and IRF (ΔRMSE = −10.47) methods produced better error-based recovery indices than the MS method. Also, the 3PL model resulted in marginally higher correlations (Δ*r* = 0.01). Again, higher guessing probability strongly reduced recovery quality (Δ*r* = −0.06 and ΔRMSE = 3.38 for an increase of Δ*c* = 0.10). Contrary to our hypotheses, increasing anchor item proportion reduced correlation of the item difficulty (Δ*r* = −0.02 when doubling the anchor item proportion), although it improved error-based recovery indices (ΔRMSE = −4.24). Furthermore, test set size had no significant effect. Larger sample sizes enhanced recovery quality of item difficulties (Δ*r* = 0.02 and ΔRMSE = −4.75 when doubling sample size). There was an ambivalent interaction between sample size and anchor item proportion: with larger samples, increasing the anchor item proportion more strongly improved correlations, *b*_*r*_ = 0.03, *t*_(392.92)_ = 6.44, *p* < 0.001, but less strongly improved the error-based indices, *b*_*RMSE*_ = 4.20, *t*_(286.45)_ = 3.29, *p* = 0.002. Increasing the discrimination parameter of the anchor items enhanced correlations strongly (Δ*r* = 0.10 for an increase of Δ*M*_*a*_ = 0.10), but not the error-based recovery indices.

### 3.4 Effects on ability parameters

Switching from MS method to the IRF or TRF method improved the correlation between true and estimated ability parameters substantially (Δ*r* = 0.06 | 0.05), whereas switching to the MM or MGM method reduced error-based recovery indices (ΔRMSE = −17.38 | −15.22). Ability parameters were recovered nearly equally well under the 2PL and 3PL models, while lower guessing probabilities again enhanced recovery quality (Δ*r* = −0.04 for Δ*c* = 0.10). Increasing anchor item proportion, test set size, and sample size improved recovery (Δ*r* = 0.01 | 0.07 | 0.01 and ΔRMSE = −10.44 | – | −5.20 when doubling the anchor item proportion | test set size | sample size). There was an interaction between the sample size and anchor item proportion: with larger samples, the negative effect of an increasing anchor item proportion on error-based recovery indices diminished, *b*_*RMSE*_ = 11.07, *t*_(207.16)_ = 4.23, *p* < 0.001. Higher discrimination ability of anchor items improved both association-based and error-based recovery indices (Δ*r* = 0.05 and ΔRMSE = −8.87 for Δ*M*_*a*_ = 0.10). Table 7 summarizes the detailed predictor effects for each parameter recovery index.

**Table 7:** Effects of the predictors on parameter recovery.

**Predictor**	**Parameter**	***r***	**Bias**	**RMSE**	**MAE**	**SEE**
MM vs. MS	a	0.01	0.27	0.48	0.24	0.43
	b	−0.04	–	−7.94	−4.73	−7.23
	θ	−0.01	–	−17.38	−7.32	−14.27
MGM vs. MS	a	0.00	−0.04	−0.06	−0.04	−0.05
	b	−0.03	–	−10.43	−6.23	−9.73
	θ	0.00	–	−15.22	−8.66	−15.97
IRF vs. MS	a	−0.01	0.00	0.16	0.03	0.16
	b	−0.02	–	−10.47	−7.33	−9.12
	θ	0.06	–	20.84	−10.86	−18.64
TRF vs. MS	a	−0.01	0.07	0.31	0.12	0.29
	b	−0.03	–	–	−5.22	–
	θ	0.05	–	18.89	−9.69	−17.07
Model	a	0.07	0.83	1.17	0.66	1.11
	b	0.01	–	–	–	–
	θ	0.00	–	–	–	–
*c*	a	−0.08	0.33	0.73	0.44	0.67
	b	−0.06	–	3.38	2.19	2.97
	θ	−0.04	–	–	3.23	–
AIP	a	0.02	−0.33	−0.39	−0.36	–
	b	−0.02	–	−4.24	−2.95	−3.84
	θ	0.01	–	−10.44	−5.20	−9.45
TSS	a	0.05	−0.56	−0.84	−0.63	−0.75
	b	–	–	–	–	–
	θ	0.07	–	–	–	–
*M_*a*_*	a	0.01	0.20	0.32	0.23	0.30
	b	0.02	–	–	–	–
	θ	0.01	–	−8.87	−4.47	−7.82
*n*	a	0.11	−0.43	−0.85	−0.50	−0.79
	b	0.10	–	−4.75	−2.72	−4.36
	θ	0.05	–	−5.20	−2.87	−4.51

The table shows the effects of the several variables predicting parameter recovery indices based on the linear mixed models. MM, mean-mean method; MS, mean-sigma method; MGM, mean-geometric mean method; IRF, item response function method; TRF, test-response function method; *c*, guessing probability; AIP, anchor item proportion; TSS, test set size; *M*_*a*_, mean anchor item discrimination parameter; *n*, sample size; a, discrimination parameter; b, item difficulty; θ, ability parameter; *r*, correlation between true and estimated parameters; RMSE, root mean square error; MAE, mean absolute error; SEE, standard error of estimate. Values indicate how indices change when switching from one method (e.g., MS to MM) or model (2PL to 3PL) to another, when doubling AIP, TSS or *n*, and when increasing *c* or *M*_*a*_ by 0.10. Example: when doubling TSS from for instance 20 to 40 items, an increase by Δ*r* = 0.07 of the correlation between true and estimated ability parameters would be expected.

### 3.5 Model convergence

**Figure 2:**
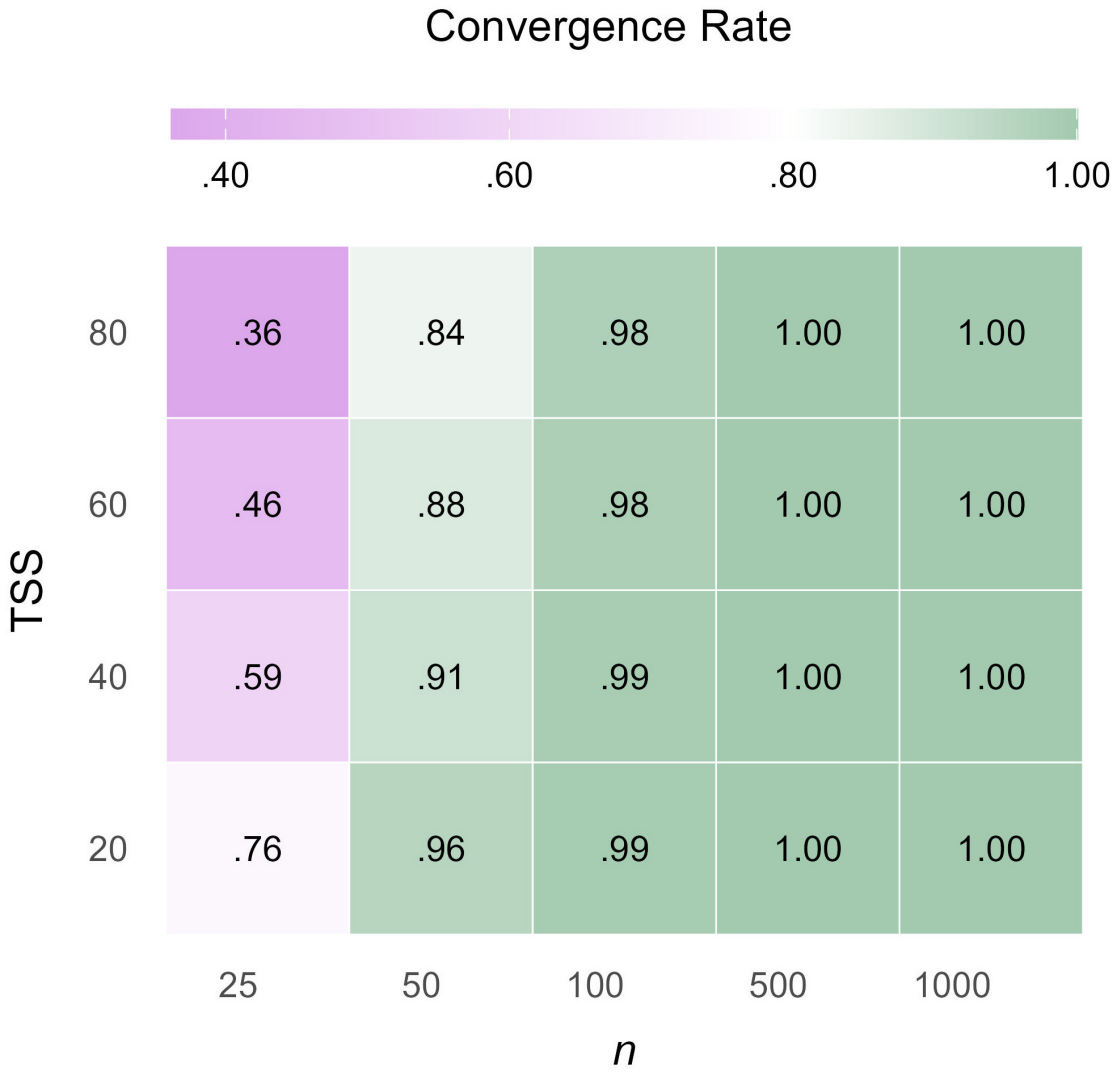
Model convergence rate depending on sample and test set size. The heatmap shows the model convergence rate depending on its two significant predictors: *n*, sample size; TSS, test set size. Convergence Rates below 0.80 (i.e., less than 80 out of 100 iterations converged) are colored in violet, above 0.80 in green.

Across all scenarios, 88.48% of the total models and on average 2.87 out of 3 test sets per estimation converged. Convergence Rates (CR) decreased by enlarging test set sizes, *b* = −0.04, *t*_(45.96)_ = −6.95, *p* < 0.001: adding 20 items per test reduced CR by 3.56 percentage points. In contrast, CR increased with increasing sample sizes, *b* = 0.09, *t*_(43.81)_ = 16.28, *p* < 0.001: adding 100 individuals per sample enhanced CR by 2.36 percentage points. The choice of IRT model, guessing probability, and anchor item proportion did not affect CR. Figure 2 displays a heatmap of CR across sample size × test set size combination: for sample sizes of *n* = 100, a minimum CR of 0.98 (i.e., on average 98 of 100 iterations converged) was observed. Also, for the number of test sets that converged, test set size (*b* = −0.06, *t*_(400.00)_ = −5.38, *p* < 0.001) and sample size (*b* = 0.10, *t*_(400.00)_ = 10.02, *p* < 0.001) were significant predictors. Additionally, marginally more test sets converged under the 2PL model than under the 3PL model (*b* = 0.00, *t*_(400.00)_ = −4.14, *p* < 0.001). Table 8 shows the detailed results of the linear mixed models predicting CR and the number of converged test sets.

**Table 8:** Results of the linear mixed model on model convergence.

**Predictor**	***b_1_***	***df***	***t***	***p***
**Convergence rate**				
Model	0.00	46.90	0.00	1.000
*c*	0.08	45.79	1.26	0.324
AIP	0.00	44.96	0.14	1.000
TSS	−0.04	45.96	−6.95	< 0.001
*n*	0.09	43.81	16.28	< 0.001
**Number of test sets converged**				
Model	0.00	400.00	−4.14	< 0.001
*c*	0.02	400.00	0.20	1.000
AIP	0.00	400.00	0.02	1.000
TSS	−0.06	400.00	−5.38	< 0.001
*n*	0.10	400.00	10.02	< 0.001

The table shows the effects of several variables predicting model convergence rate and the number of test sets converged per model estimation. *c*, guessing probability; AIP, anchor item proportion; TSS, test set size; *M*_*a*_, mean anchor item discrimination parameter; *n*, sample size; *df* , degrees of freedom. To correct for multiple testing, *p* values have been Benjamini-and-Hochberg adjusted (*q* = 0.050).

## 4 Discussion

### 4.1 Interpretation and implications of the results

The goal of this study was to provide researchers with reliable information and methods to fully utilize the potential of test equating. For this purpose, we used computer-simulated data and a parameter recovery approach to examine the extent to which key factors of test equating affect the stability of IRT parameters. Therefore, we considered the correlations between true and estimated discrimination parameters, item difficulties, and ability parameters as association-based recovery indices, and bias, RMSE, MAE, and SEE as error-based recovery indices. The overall correlation of the discrimination parameters was rather low (*r* = 0.55). In contrast, the correlation of both the item difficulties (*r* = 0.81) and the ability parameters (*r* = 0.83) were considerably higher. We employed five common equating methods (MS, MM, MGM IRF and TRF method) discussed in the literature. Indeed, the choice of the equating method had an impact on how well the IRT parameters were recovered: for discrimination parameters, the MM and MGM method led to better recovery quality, for the item difficulties the MM, MGM, and IRF method were convenient, and for ability parameters, the MS method was outperformed by all the other methods. However, the effect of the equating method was relatively small, which is in line with previous research from Battauz (2017).

In line with H2, the choice of the IRT model affected parameter stability: the 3PL model produced notably higher correlations between true and estimated ability parameters which is plausible since in contrast to the 2PL model the 3PL model takes the guessing probability into account. However, switching from 2PL to 3PL model deteriorated error-based recovery indices. This can be explained by the fact that in case of the 3PL model, the slope of the item characteristic curve is systematically underestimated, and this underestimation constitutes a linear transformation of the discrimination parameter which does not affect the correlation (e.g., Baker and Kim, 2004; Lord, 1980). To investigate the role of guessing probability, we varied the number of response options to an item. Guessing probability strongly affected the stability of all three IRT parameters: in line with H3, lower guessing probabilities resulted in higher stability, with the largest improvement when the guessing probability was eliminated entirely. This might lead to the recommendation to employ items with distractor-free response formats whenever possible to enhance parameter stability.

Regarding H4 and H5, enlarging anchor item proportion and test set size substantially improved the stability of discrimination and ability parameters. These findings are in line with Sinharay and Holland (2007) and extend previous research by examining smaller and more flexible anchor item proportions as well as larger test set sizes, enhancing the generalizability of these effects. Furthermore, we could show that the effects of enlarging anchor item proportion and test set size on IRT parameter stability are not linear but approximatively logarithmic: there was a diminishing marginal utility in terms of a saturation effect which means that small item sets benefit more from additional items than larger item tests. Additionally, in line with H6, our results complement existing research by indicating that the benefit of increasing anchor item proportion on the stability of the discrimination parameters decreased as test set size increased. This interaction and the diminishing marginal utility effect may guide important practical implications: in settings where resources are limited, test set size and, as a consequence, the duration of the assessment can be reduced, while this reduction can be compensated to some degree by augmenting the anchor item proportion in order to get reasonable estimates of discrimination and ability parameters. In contrast to discrimination and ability parameters, test set size had no significant effect on the stability of the item difficulties, and the influence of the anchor item proportion was ambiguous: increasing anchor item proportion reduced correlations but improved error-based indices. One explanation for this ambiguous effect might be variance shrinkage of anchor item difficulty: in small anchor item sets, the variance of (true) item difficulties is statistically higher than in larger anchor item sets which can reduce the correlation slightly. However, error-based recovery indices remain unaffected by this shrinkage since they do not depend on rank-orders but on absolute deviations. Prior work suggests that homogenizing anchor item difficulties toward the center of the difficulty scale enhances parameter estimation (e.g., Dorans et al., 2007; Sinharay et al., 2012).

In line with H7, higher discrimination parameters of the anchor items strongly increased the stability of all three IRT parameters. These findings correspond with research on DIF detection (e.g., Lopez Rivas et al., 2009; Meade and Wright, 2012) and extend the benefit of high-discrimination items to test equating contexts.

According to H8, enlarging the sample size had a strong effect on the recovery of all three IRT parameters, particularly on the recovery of discrimination parameters and item difficulties. For discrimination parameters, association-based recovery profited especially from enlarging the sample from *n* = 100 (*r* = 0.47) to *N* = 500 (*r* = 0.73) while error-based recovery indices were already relatively low at *n* = 100 individuals. For item difficulties and ability parameters, the pattern is inversed: samples of *n* = 100 provided high association-based recovery indices, but for acceptable error-based recovery indices larger sample sizes with a minimum of *n* = 500 are necessary. These sample size thresholds might be relevant for item banking where it is aimed to create large banks of items with stable item characteristics but where it is necessary to consider personnel and temporal resources. Consistent with H9, increasing sample size reduced the number of anchor items required to achieve high stability of discrimination and ability parameters. This effect might hold practical implications for periodically recurring assessments such as PISA (OECD, 2019) or annual student selection tests, where a small number of anchor items is advantageous since it reduces the probability of anchor item leakage which would bias ability estimation.

Model CR strongly depended on sample size and test set size: Small samples of 25 or 50 individuals were particularly prone to converge failures, especially when employing tests with many items. This is in line with best-practice recommendations on IRT modeling which suggest samples of at least 500 (2PL) or 1,000 (3PL) individuals. However, results indicate that under certain conditions these thresholds may be relaxed to sample sizes of 100 individuals: in low-stakes settings, where individual test results are less consequential and the primary interest lies in detecting general trends, a 1–2% risk of non-convergence may be justifiable. In such cases, more constrained models (e.g., fixed guessing probability or 1PL model) or the exclusion of items with poor statistical properties could help maintain adequate stability. In contrast, in high-stakes settings (e.g., student selection tests) where test results have crucial consequences for participants, ensuring model convergence is essential. Consequently, when different test forms are administered in such contexts, it should be considered that each form is completed by a sufficient number of participants (i.e., *n* ≥ 500).

In order to facilitate researchers to gauge which stability can be expected from a specific factor level combination we provide our data and the *R* code on the OSF. For individual test development and experimental design purposes, we further provide an instruction for examining the parameter stability under arbitrary conditions that are not included in this article. The *R* simulation code is prepared in a way that allows (1) the ability distributions of the samples, (2) the sample sizes, (3) the number of test sets, (4) the number of items, (5) the anchor item proportion, (6) the number of iterations, (7) the IRT model, (8) the guessing probability, and (9) the equating method to be varied easily. The *R* simulation code and instructions on how to use the code are available on our OSF: https://osf.io/np7k9/?view_only=dcb2b2b1bb02426e95b882f48378aa81.

### 4.2 Conclusion

These considerations lead to the following three recommendations: first, to enhance the stability of all three IRT parameters, guessing probability should be minimized, anchor items should have high discrimination parameters, and larger sample sizes should be used: while sample sizes of 25 of 50 produce inacceptable estimates, in some cases samples of already 100 or 500 individuals may be justifiable. However, although acceptable convergence rates were observed with samples of 100 individuals, model convergence is not guaranteed when sample sizes fall below 500. Second, to improve the stability of discrimination and ability parameters in particular, large test sets or high anchor item proportions should be applied. Importantly, augmenting one of these two factors can compensate to some extent for reductions in the other one. Third, although certain equating methods perform slightly better for specific IRT parameters, the overall advantages are comparatively small.

## Data Availability

The datasets presented in this study can be found in online repositories. The names of the repository/repositories and accession number(s) can be found at: https://osf.io/np7k9/?view_only=dcb2b2b1bb02426e95b882f48378aa81.
